# The Glutaminase-Dependent System Confers Extreme Acid Resistance to New Species and Atypical Strains of *Brucella*

**DOI:** 10.3389/fmicb.2017.02236

**Published:** 2017-11-15

**Authors:** Luca Freddi, Maria A. Damiano, Laurent Chaloin, Eugenia Pennacchietti, Sascha Al Dahouk, Stephan Köhler, Daniela De Biase, Alessandra Occhialini

**Affiliations:** ^1^Institut de Recherche en Infectiologie de Montpellier, Centre National de la Recherche Scientifique, Institut National de la Santé et de la Recherche Médicale, Université de Montpellier, Montpellier, France; ^2^Department of Medico-Surgical Sciences and Biotechnologies, Sapienza University of Rome, Laboratory Affiliated to the Istituto Pasteur Italia – Fondazione Cenci Bolognetti, Latina, Italy; ^3^German Federal Institute for Risk Assessment Berlin, Germany

**Keywords:** *Brucella*, acid resistance, extreme acid stress, glutaminase, glutamate decarboxylase, GadC antiporter

## Abstract

Neutralophilic bacteria have developed specific mechanisms to cope with the acid stress encountered in environments such as soil, fermented foods, and host compartments. In *Escherichia coli*, the glutamate decarboxylase (Gad)-dependent system is extremely efficient: it requires the concerted action of glutamate decarboxylase (GadA/GadB) and of the glutamate (Glu)/γ-aminobutyrate antiporter, GadC. Notably, this system is operative also in new strains/species of *Brucella*, among which *Brucella microti*, but not in the “classical” species, with the exception of marine mammals strains. Recently, the glutaminase-dependent system (named AR2_Q), relying on the deamination of glutamine (Gln) into Glu and on GadC activity, was described in *E. coli*. In *Brucella* genomes, a putative glutaminase (*glsA*)-coding gene is located downstream of the *gadBC* genes. We found that in *B. microti* these genes are expressed as a polycistronic transcript. Moreover, using a panel of *Brucella* genus-representative strains, we show that the AR2_Q system protects from extreme acid stress (pH ≤2.5), in the sole presence of Gln, only the *Brucella* species/strains predicted to have functional *glsA* and *gadC*. Indeed, mutagenesis approaches confirmed the involvement of *glsA* and *gadC* of *B. microti* in AR2_Q and that the acid-sensitive phenotype of *B. abortus* can be ascribed to a Ser248Leu substitution in GlsA, leading to loss of glutaminase activity. Furthermore, we found that the gene BMI_II339, of unknown function and downstream of the *gadBC–glsA* operon, positively affects Gad- and GlsA-dependent AR. Thus, we identified novel determinants that allow newly discovered and marine mammals *Brucella* strains to be better adapted to face hostile acidic environments. As for significance, this work may contribute to the understanding of the host preferences of *Brucella* species and opens the way to alternative diagnostic targets in epidemiological surveillance of brucellosis.

## Introduction

*Brucella*, a non-motile Gram-negative intracellular facultative coccobacillus, is the causative agent of brucellosis, the most widespread bacterial zoonosis, infecting livestock and humans (human incidence: 500,000 cases/year). Brucellae transmission routes to humans include direct contact with infected animal tissues, inhalation of airborne bacteria, and most frequently, ingestion of contaminated and unpasteurized dairy products (Pappas et al., 2006).

*Brucella* exists as a genus since 1920 (Mayer and Shaw, 1920); to date, it comprises 12 species of which the most recently published as such is *Brucella vulpis* (Scholz et al., 2016). *Brucella* spp. have very similar genomes and are classified essentially on the basis of their host preference, pathogenicity, and a number of biochemical and phenotypic traits. Six species, isolated and described at least 20 years ago, are considered as “classical”; they occur in terrestrial mammals: *Brucella abortus* in cattle, *Brucella melitensis* and *Brucella ovis* in small ruminants, *Brucella suis* in pigs, *Brucella canis* in dogs, and *Brucella neotomae* in desert wood rats. Two species have been isolated from marine mammals: *Brucella ceti* from cetaceans and *Brucella pinnipedialis* from pinnipeds. Human brucellosis has been reported to be caused by *B. abortus*, *B. melitensis*, and *B. suis* biovars 1–4 (Pappas et al., 2006). More recently *B. canis*, marine species and *B. neotomae* have been associated to human cases of brucellosis (Sohn et al., 2003; Marzetti et al., 2013; Suárez-Esquivel et al., 2017).

In the past decade new species have been described, such as *Brucella microti* isolated from common vole and *Brucella inopinata* from humans (Scholz et al., 2008, 2010). Atypical strains of *Brucella*, from Australian rodents, amphibians, and ray have also been isolated (Tiller et al., 2010; Soler-Lloréns et al., 2016; Al Dahouk et al., 2017; Eisenberg et al., 2017). Except for, *Brucella papionis*, isolated from baboons (Whatmore et al., 2014), *B. vulpis* from red fox (Scholz et al., 2016), and strains from Australian rodents, the newly described species and atypical strains are metabolically more active and grow faster than the classical species. This suggests that they have a more versatile metabolism that could favor survival in the environment (Aujoulat et al., 2012; Al Dahouk et al., 2017).

Since 2001, *B. microti* has been isolated in Central Europe from soil and wild animals such as *Microtus arvalis* (i.e., the common vole), red fox, and wild boar (Scholz et al., 2008, 2009; Rónai et al., 2015). In addition to the rapid replication in murine and human macrophage cells, *B. microti* is the first species of *Brucella* described to be lethal in experimentally infected mice by intraperitoneal injection (Jiménez de Bagüés et al., 2010). Moreover, this species is more acid-tolerant than *B. suis* in a synthetic minimal medium at a pH of 4.5, i.e., mimicking the acidity encountered by *Brucella* in the host cell phagolysosome.

In *B. microti*, the glutamate decarboxylase (Gad)-dependent system (also named AR2) was shown to play an important role in acid resistance (AR) not only in a synthetic minimal medium at pH 2.5, but also in the experimental infections of mice via oral route (Occhialini et al., 2012). In many orally acquired bacteria (such as *Escherichia coli*, *Shigella flexneri*, *Listeria monocytogenes*, *Lactococcus lactis*, and *Lactobacillus reuteri*), the Gad system is the most effective among all the amino acid-dependent AR systems (Lund et al., 2014). Briefly, upon exposure to an extremely low pH, the Gad system becomes active: a molecule of L-glutamate (Glu) is taken up *via* the inner membrane antiporter GadC and decarboxylated into γ-aminobutyrate (GABA) by the cytosolic enzyme Gad; GABA is then exported via GadC in exchange for a new Glu molecule. Overall, the Gad system provides protection from extreme acid stress because the Gad enzyme consumes an intracellular proton at each decarboxylation cycle, while the antiporter GadC operates a net export of positive charges, thereby contributing to the maintenance of the membrane potential (Richard and Foster, 2004; De Biase and Pennacchietti, 2012).

More recently the antiporter GadC and the enzyme glutaminase (YbaS in *E. coli* and Gls3 in *L. reuteri*) were shown to act in concert and to constitute a novel and alternative AR system based on the deamination of L-glutamine (Gln or Q), one of the most abundant free amino acids (Lu et al., 2013; Teixeira et al., 2014). This system was therefore named AR2_Q because it utilizes GadC, which is part of the AR2 system, to exchange an extracellular Gln molecule with an intracellular Glu (or GABA) molecule (Lund et al., 2014). The enzyme glutaminase converts Gln into Glu by releasing ammonia (NH_3_), which at acidic pH remains in solution and is fully protonated as ammonium ion (${NH}_{4}^{+}$), and in addition to this can also feed intracellularly the Gad system with a Glu molecule which can be further decarboxylated into GABA by GadB. Thus, the Gad- and Gln-dependent systems can also cooperate in achieving intracellular pH neutralization more efficiently.

Our group showed that in *B. microti gadB* and *gadC* constitute a functional Gad system homologous to that of *E. coli* (Occhialini et al., 2012). Intriguingly, this system is functional in practically all the new species and atypical strains as well as in those isolated from marine mammals, but is not functional in the classical terrestrial *Brucella* species (Occhialini et al., 2012; Damiano et al., 2015). Notably, the *gadB* and *gadC* genes in the chromosome II of all sequenced strains of *Brucella* are located immediately upstream and in the same orientation of a putative glutaminase-encoding gene (*glsA*), the *hdeA* gene, encoding a chaperone involved in AR in *E. coli* and in *B. abortus* (Gajiwala and Burley, 2000; Valderas et al., 2005), and two genes with unknown function.

Using physiological, genetic, and molecular approaches, this work was undertaken with the aim of (i) assessing the existence of the AR2_Q system in *B. microti*; (ii) establishing whether the genes potentially encoding the AR2 and AR2_Q systems (*gadB*, *gadC*, *glsA*) form an operon with *hdeA* and the two downstream genes of unknown function; (iii) studying the role of each of these genes in extreme AR; and (iv) comparing the Gln-dependent AR phenotypes within the *Brucella* genus.

## Materials and Methods

### Bacterial Strains and Culture Conditions

Twenty-six reference strains of *Brucella*, including *B. microti* CCM4915, were studied (Supplementary Table S1). The Δ*gadB*- and Δ*gadC*-mutant strains of *B. microti* previously constructed were added as controls (Occhialini et al., 2012). The *E. coli* K12 strains DH5α and MG1655 were used for cloning purposes and plasmid production. The strains of *Brucella* and *E. coli* were grown at 37°C in Tryptic Soy (TS, Difco) and Luria Bertani (LB, Becton-Dickinson) broth, respectively. *B. abortus*, *B. neotomae*, *B. ovis*, *B. ceti*, and *B. pinnipedialis* were cultured in a 5% CO_2_ atmosphere and, only for the latter two species, by supplementing the TS broth with 10% fetal calf serum. Derivative mutant strains of *B. abortus* and *B. microti* were also created as described below. When appropriate, media were supplemented with kanamycin or ampicillin at 50 μg/ml, or with chloramphenicol at 30 μg/ml.

### Amino Acid-Dependent Acid Survival Assays

Acid survival assays of wild-type and mutant strains described hereafter were performed with bacteria grown for 24–26 h in TS broth (final pH 7.0), as reported in previous studies aimed at establishing the functionality of the Gad system (Occhialini et al., 2012; Damiano et al., 2015). Briefly, comparative analyses of extreme acid survival were carried out by 1:500-dilution of the inoculum from the 24-h cultures in modified Gerhardt’s medium (GMM without Glu) with or without 3 mM of each amino acid (Sigma Aldrich) and brought to different pHs (2.5 or 7) with HCl. At the indicated time points, aliquots (20 μl) were withdrawn, diluted (1:10) in GMM pH 7.0, and plated (100 μl) onto TS agar plates to enumerate the colony-forming units (CFU), corresponding to viable bacteria. All assays were performed at least in triplicate.

Because TS contains 0.25% glucose, these conditions closely resemble those (buffered LB, supplemented with 0.4% glucose) employed by Lu et al. (2013) to discover the AR2_Q system in *E. coli*.

### Construction and Complementation of *Brucella*-Mutant Strains

Each mutant strain of *B. microti* (Δ*gadB/C-*Δ*glsA*, Δ*glsA*, Δ*hdeA*, ΔBMI_II338, and ΔBMI_II339) was obtained by replacing the target gene(s) with a kanamycin resistance cassette, devoid of a transcription terminator, obtained by PCR amplification from plasmid pUC4K as template. To generate mutant strains, a recombinant plasmid derived from pGEM-T Easy (non-replicative in *Brucella*) was first constructed in *E. coli* DH5α and then introduced into *B. microti* by electroporation, as described elsewhere (Hanna et al., 2011). To select for allelic exchange mutants, the Kan*^R^* colonies were checked for sensitivity to ampicillin, carried by pGEM-T. Homologous exchange in isolated Kan*^R^*/Amp*^S^* clones was validated by PCR. Homologous complementation of mutant strains of *B. microti* was obtained by transformation with the shuttle vector pBBR1-MCS carrying the complete sequence of the corresponding deleted ORF (*glsA*, *gadB/C*, *gadC-glsA*, BMI_II338, and BMI_II339), using a strategy reported elsewhere (Occhialini et al., 2012). Briefly, each DNA fragment was obtained by PCR amplification using a high fidelity *Pfx* polymerase (Life Technologies) and then directionally inserted in the pBBR1-MCS plasmid at *Xho*I*/Xba*I restriction sites, in the orientation of the *lacZ* gene (Occhialini et al., 2012; Damiano et al., 2015). All PCR primers used for the genetic manipulations are listed in Supplementary Table S2.

Site-directed mutagenesis on *B. abortus glsA* was performed by overlap extension polymerase chain reactions, following the procedure described by Heckman and Pease (2007). Briefly, segments of the target gene are amplified from the genomic DNA of *B. microti* as template using two flanking master primers (Master_glsA_XhoI_For and Master_glsA_XbaI_Rev) that mark the 5′-ends of both strands and two pairs of internal primers for each of the two mutations Phe61→Ile (M61-glsA-Union-For/Rev) and Ser248→Leu (M248-glsA-Union-For/Rev) of interest. The flanking master primers (Master_glsA_XhoI_For and Master_glsA_XbaI_Rev) were then used to fuse the two PCR fragments carrying either Ser248→Leu or Phe61→Ile mutation into a new *glsA* ORF carrying either of the mutation. Plasmids pBBR1-MCS_*glsA*_Phe61Ile and pBBR1-MCS_*glsA*_Ser248Leu were sequenced on both strands and subsequently used to transform *B. abortus*.

The *E. coli ybaS* gene was manufactured and cloned at the Eurofins Genomics division in Ebersberg, Germany^1^. Briefly, the 1121-bp synthetic sequence included the 933-bp *ybaS* ORF, the 156-bp upstream promoter region, the 20-bp 3′-non-coding region, and 6-bp *Xho*I (at the 5′-end) and 6-bp *Xba*I (at the 3′-end) restriction sites. The synthetic gene was checked by double-strand DNA sequencing (Eurofins Genomics DNA sequencing facility). The sequence was found to be 100% congruent and subsequently cloned into plasmid pBBR1-MCS (also this fully sequenced by the same service), linearized with the same restriction enzymes as above, to allow directional cloning respect to the *lac* promoter in pBBR1-MCS.

### Gene Expression Analysis of the *gadB/C–glsA–hdeA Locus* by RT-PCR and RNAseq

Total RNA of *B. microti* was extracted from cultures at the stationary-phase of growth in TS broth pH 7.0 at 37°C with shaking. Gene expression profiles were frozen by immediate addition of a phenol/ethanol mix (10% phenol) at 1/10 of the final volume. Total RNAs were extracted and purified using the mirVana Kit (Ambion), followed by spectrophotometric quantification (NanoDrop Technologies) and Agilent chip quality control (Agilent Technologies). To eliminate contaminating DNA, RNA was treated with RNase-free DNase (Turbo DNA, Ambion).

To determine the operon organization of the genes of the *gad* locus, two primers were designed to hybridize at the 3′-end of the upstream gene and the 5′-end of the downstream gene for each pair of consecutive genes, so that a fragment was amplified by RT-PCR (Supplementary Table S2). For production of cDNA, 1 μg of total RNA was retro-transcribed using a 6-mer random primer mix and the Superscript II Reverse Transcriptase enzyme (Life Technologies) in a reaction volume of 20 μl at 42°C for 2 h; then, cDNA was diluted at 1/20 and 2 μl used in a 50 μl PCR reaction. PCR amplification was carried out using 1 unit of GoTaq polymerase (Promega), 2 mM MgCl_2_, 200 μM of each dNTP, 25 μM of each primer, with 35 cycles at: 94°C for 30 s, 54°C for 30 s, and 72°C for 30 s to 1 min (according to the expected size of the products). Two nanograms of genomic DNA of *B. microti* was used as a positive control and water as negative control of the reaction. For each RNA sample, the absence of genomic DNA contaminants was verified by using not retro-transcribed RNA as template in the PCR reactions. Products were separated on an agarose gel (1.2%) and stained with SYBR Safe (Life Technologies).

Expression levels of the genes in the *gad locus* were also studied by RNAseq analysis performed by GATC Biotech AG (Constance, Germany), using the Illumina TruSeq protocol consisting in depletion of rRNAs and degraded mRNAs, construction of a random-primed cDNA library and subsequent sequencing on Illumina’s High Seq 2000 in single read mode with a read length of 50 bases. The obtained reads were analyzed after mapping against the genomic reference of *B. microti* CCM4915.

### Bioinformatic and Modeling Studies

The bioinformatic tools available at the ExPASy website^2^ were used to predict the secondary structure and the possible occurrence of transmembrane domains and of a signal peptide sequence in BMI_II339. The results reported herein were obtained using the TMHMM software (available at www.cbs.dtu.dk/services/TMHMM/). Protein sequences alignments were generated with Clustal Omega (version 1.2.4).

The glutaminases model structures from *Brucella* were obtained by comparative homology modeling using Modeller v9.18 (Sali and Blundell, 1993) and using several glutaminases as template structures (PDB codes: 3AGD, 4BQM, 3IF5, 3IH8, 1MKI, 2OSU, 2PBY, 3SS3, 1U60, 3UO9, 3VOY).

### Statistical Analysis

Data from AR assays were analyzed via “two-way ANOVA” using the Bonferroni test (as available in the GraphPad Prism software suite, version v5.0a). Data were expressed as means of three independent experiments with standard deviations. In order to perform the ANOVA analysis, all data were converted into percentage of survival for each time-point (30, 60, and 120 min) (*n* = 3 per group) with respect to time 0 (set as 100%). Differences were considered statistically significant when *P* < 0.05.

## Results

### The Presence of Glutamine or Glutamate Is Sufficient to Confer Extreme Acid Resistance to *B. microti*

**FIGURE 1:**
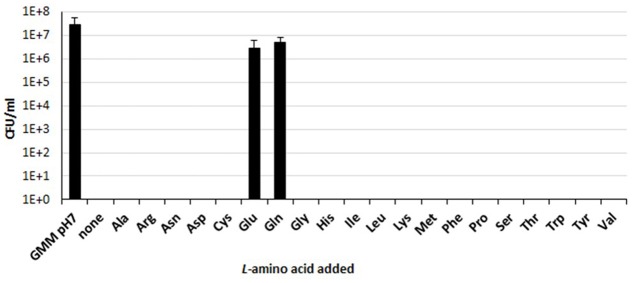
Amino acid-dependent survival of *B. microti* CCM4915 after acid exposure. Bacteria from a stationary-phase culture in TS at pH 7 were harvested and incubated in modified GMM at pH 7 (first column, GMM pH 7) or at pH 2.5, either without (none) or with 3 mM of each of the indicated amino acids. Residual viability at 30 min was expressed as CFU/ml on a log10-scale. The data represent the mean (SD) of three independent experiments.

Our group has previously demonstrated that a functional Gad system, relying on Glu supplementation, confers extreme AR to *B. microti*, marine mammal species, and other new and atypical strains of *Brucella* (Occhialini et al., 2012; Damiano et al., 2015). To explore the occurrence of other amino acid-dependent AR systems, the survival rates of *B. microti* were determined *in vitro* using the minimal medium GMM at pH 2.5 individually supplemented with 1 of the 20 proteinogenic amino acids. Under these experimental conditions, only Glu and Gln were found to confer protection from extreme acid stress (**Figure 1**). In fact, while after 30 min of exposure to extreme acid conditions and in the presence of either of these two amino acids, the number of viable bacteria decreased by less than 10-fold (from 5 × 10^7^ to ≥5 × 10^6^ CFU/ml), no survivors were found in the presence of any of the other amino acid tested or in their absence (**Figure 1**). These results demonstrated that the presence of either Gln or Glu was sufficient to protect *B. microti* from extreme acid stress and that this species, in addition to the Gad system (Occhialini et al., 2012; Damiano et al., 2015), may have a functional glutaminase-based (AR2_Q) system. A bioinformatics analysis was thus undertaken to search for AR2_Q candidate genes in *B. microti*.

### The *gadB*, *gadC*, *glsA*, and *hdeA* Genes of *B. microti* Are Co-transcribed

**FIGURE 2:**
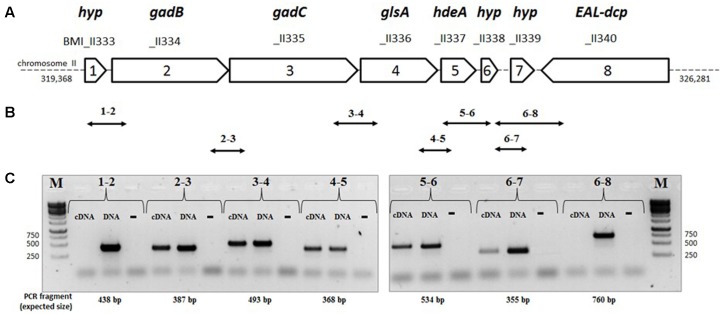
Organization of the genes of the *gadB/C–glsA* locus of *B. microti* CCM4915. **(A)** Map of the *gad-EAL-dcp* region: each arrow represents an ORF, with the reported length proportional to its size on chromosome II of *B. microti*. Gene name: *gadB* (Glu decarboxylase), *gadC* (Glu/GABA antiporter), *glsA* (glutaminase), *hdeA* (chaperone protein HdeA), *EAL-dcp* (EAL-*d*omain *c*ontaining *p*utative protein). The corresponding locus designations (locus tags) are shown above each arrow. **(B)** Double-headed arrows indicate the positions and sizes of the expected intergenic PCR fragments. **(C)** Agarose gel electrophoresis of the RT-PCR products. For each pair of primers, indicated by pair of numbers, three separate PCR reactions were performed using as template cDNA (test reaction), genomic DNA (“DNA,” positive control), and not-reverse transcribed RNA of *B. microti* (“-”, negative control). *M* = 1 kb DNA ladder (Euromedex). The sizes of relevant ladder bands are shown on the left and on the right of each panel, respectively.

Analysis of the genome sequence of *B. microti* reveals that, unlike *E. coli* that possesses two glutaminase-encoding genes (*ybaS* and *yneH*) (Lu et al., 2013), this species possesses only one gene encoding a putative glutaminase, namely *glsA* (BMI_II336). The *glsA* gene is located on chromosome II immediately downstream the *gadB/C* locus (BMI_II334 and BMI_II335), and upstream the *hdeA* gene (BMI_II337), encoding the Gad system structural components and the HdeA periplasmic chaperone, respectively (**Figure 2A**). The latter was shown to play a role in resistance to acid stress in *E. coli* and *B. abortus* (Gajiwala and Burley, 2000; Valderas et al., 2005). Three other genes encoding hypothetical proteins are placed in the same orientation as *gadBC*, *glsA*, and *hdeA*, namely BMI_II333 and BMI_II338/BMI_II339, upstream of *gadB* and downstream of *hdeA*, respectively (**Figure 2A**). This prompted us to investigate whether all these genes are part of a polycistronic operon.

To assess this, RT-PCR analyses were performed using cDNA as template and pairs of primers between consecutive genes. The strategy is depicted in **Figure 2B**. PCR fragments of the expected sizes were obtained from the cDNA template, except for pairs 1–2 and 6–8 (**Figures 2B,C**), thus indicating that the operon extends from BMI_II334 to BMI_II339 (herein named *gadBC*–*glsA* operon) and therefore does not include BMI_II333 and, as expected, BMI_II340. The lack of amplification using RNA not reversed-transcribed into cDNA proves the absence of genomic DNA as contaminant.

The presence of a polycistronic operon was corroborated by RNA-seq analysis which also indicated the existence of additional transcription start sites upstream of *hdeA* and BMI_II339, respectively (Supplementary Figure S1). This indicated that the latter genes can also be independently transcribed from *gadBC–glsA*.

### Genes of the *gadBC–glsA* Operon of *B. microti* Participate in Extreme Acid Stress Resistance

**FIGURE 3:**
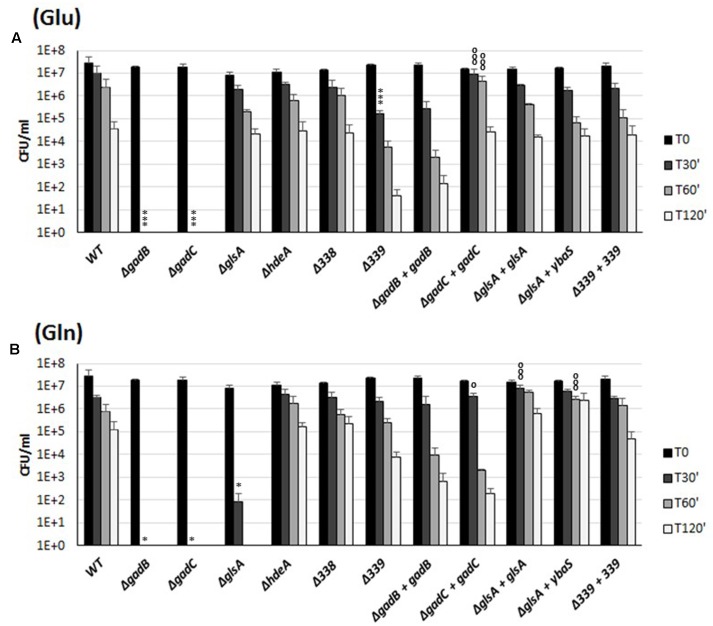
Survival of the wild-type and mutant strains of individual genes of the *B. microti* CCM4915 *gadBC*–*glsA* operon during extreme acid exposure. Bacteria from stationary-phase cultures in TS at pH 7 were harvested and incubated in modified GMM at pH 2.5, supplemented with 3 mM of Glu **(A)** or Gln **(B)**. Mutant strains are indicated by “Δ” and the designation of the inactivated gene. Complemented mutant strains are designated by “+” followed by the name of the *in trans* complementing gene. Residual viability following the acid shock was expressed as CFU/ml on a log10-scale at each time-point (T30, T60, and T120 min) and compared to unchallenged bacteria present at time T0 (GMM, pH 7.0). No viable bacteria were detected at the same time-points in the absence of Glu or Gln (data not shown). The data represent the mean (SD) of three independent experiments. A two-way ANOVA with a Bonferroni post-test statistical analysis was performed between wild-type and each mutant strain (significance ^∗^*P* < 0.05, ^∗∗^*P* < 0.01, ^∗∗∗^*P* < 0.001) and between each mutant strain and the corresponding complemented strain (significance °*P* < 0.05, ^∘∘^*P* < 0.01, ^∘∘∘^*P* < 0.001).

The co-expression of the genes of the *gadBC*–*glsA* operon and the occurrence of a Glu- and Gln-dependent AR in *B. microti* (**Figures 1**, **2**) suggested the involvement of the relevant proteins in a common biological function, i.e., conferring resistance to extreme acid stress. To investigate this hypothesis, the role of the individual genes in extreme AR in the presence of either Glu or Gln was studied by analyzing the AR phenotypes of the mutants and complemented strains. Each mutant strain was constructed by inactivation of the corresponding gene following insertion of a Kan*^R^* cassette devoid of a transcriptional terminator, while the complemented strains were obtained by expressing *in trans* an intact copy of the relevant gene cloned into plasmid pBBR1-MCS. The ability of these strains to resist to extreme acid stress was therefore assessed by determining their viability following incubation in modified GMM at pH 2.5, with and without Glu or Gln, respectively (**Figure 3**).

Data in **Figure 3A** confirm that the *gadB* and *gadC* genes encode the structural components of the Gad system (Occhialini et al., 2012), and also show participation of BMI_II339 in the Glu-based AR.

The Gln-dependent AR phenotype of *B. microti* was almost completely abolished (i.e., 5.5 log10 reduction) in the Δ*glsA* mutant strain already after 30 min of acid challenge, while the complementation with the intact *glsA* gene of *B. microti* or *ybaS* gene of *E. coli* restored the AR2_Q phenotype even to a better extent than in the wild-type strain, probably due to their expression from a multicopy plasmid (**Figure 3B**). These results provide evidence that GlsA of *Brucella* and YbaS of *E. coli*, which share 61.8/75.7% amino acid identity/similarity, are functionally homologous enzymes. In addition to this, the phenotypes of the Δ*gadC*-mutant strain and of its complemented derivative in the presence of Gln strongly suggest that both *glsA* and *gadC* are the molecular determinants of the AR2_Q system. The acid-sensitive phenotype of the Δ*gadB-*mutant strain in the presence of Gln (**Figure 3B**) indicated that the insertion of the Kan*^R^* cassette, despite the absence of a terminator sequence, exerts an unexpected polar effect on the expression of downstream *gadC* and *glsA* (as confirmed by RT-qPCR analysis; data not shown).

In the presence of either Glu or Gln, the survival of the Δ*hdeA-* and ΔBMI_II338-mutant strains of *B. microti* was similar to that of the wild-type strain (**Figure 3**). Therefore, under our experimental conditions, a contribution of the HdeA chaperone and of the putative product of the BMI_II338 gene to the amino acid-dependent AR phenotype of *B. microti* at the extreme pH of 2.5 can be ruled out. As mentioned above, the last gene in the operon, BMI_II339, when deleted caused a noticeable decrease in AR ( ≈2.5 log10 and ≈1.5 log10 after 2 h, as compared to the wild-type strain, in the presence of Glu and Gln, respectively), suggesting that this gene may play a role in the AR phenotype.

**FIGURE 4:**
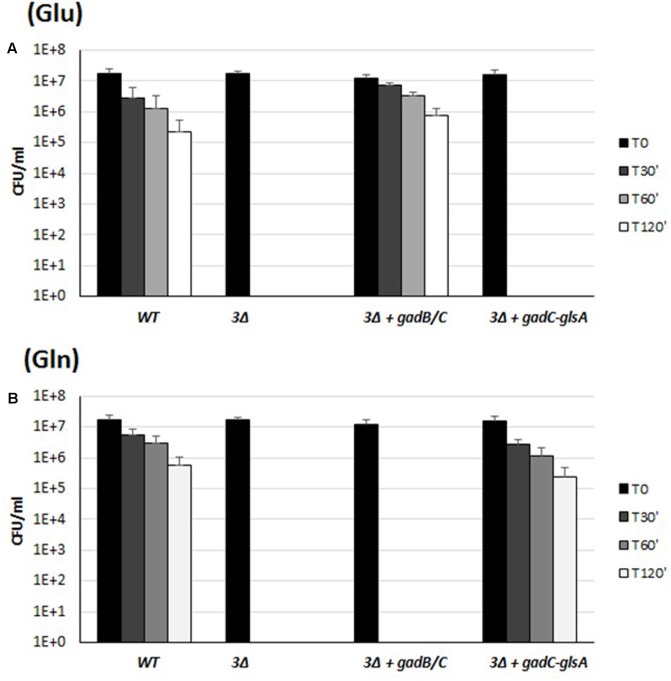
Survival of the wild-type, the triple mutant *gadB/C*–*glsA* and the complemented mutant strains of *B. microti* CCM4915 during extreme acid exposure. Bacteria from stationary-phase cultures in TS at pH 7 were harvested and incubated in modified GMM at pH 2.5, supplemented with 3 mM of Glu **(A)** or Gln **(B)**. In the triple mutant strain, indicated as “3Δ,” *gadB/C*–*glsA* genes were replaced by a Kan*^R^* resistance cassette. Mutant strains complemented for the Gad system or the Gln system are indicated with “+” followed by the designation of the relevant genes. Residual viability following the acid shock was expressed as CFU/ml on a log10-scale at each time-point (T30, T60, and T120 min) and compared to unchallenged bacteria present at time T0 (GMM, pH 7.0). No viable bacteria were detected at the same time-points in the absence of Glu or Gln (data not shown). The data represent the mean (SD) of three independent experiments.

Several genetic approaches based on counter-selectable marker systems such as *sacB* and *galK* (Dozot et al., 2006; Barkan et al., 2011) were attempted to obtain specific and non-polar mutant strains of *B. microti*, but without success. Thus, to evaluate the relative contribution of the pair of genes *gadB/C* and *gadC–glsA* to extreme AR, we created a triple mutant strain Δ*gadB/C-*Δ*glsA* (indicated in **Figure 4** as “3Δ”), and partially complemented it *in trans* with either the *gadB/C* or the *gadC–glsA* pair of genes, cloned into plasmid pBBR1-MCS. As shown in **Figure 4**, Glu confers AR to strains expressing the GadB and GadC (**Figure 4A**), whereas Gln confers AR only to the strains expressing GadC and GlsA (**Figure 4B**). This strategy allowed us to conclude, that in the presence of Glu and Gln, the AR2 (Gad system) and the AR2_Q (glutaminase system), respectively, of *B. microti* individually confer bacterial resistance during exposure to a strongly acidified medium. On the other hand, the acid sensitive phenotype in the presence of Gln or Glu of the triple-mutant strain complemented with *gadB/C* or *gadC*–*glsA*, respectively, can be ascribed only to the lack of AR2_Q and Gad systems, and it is not a consequence of a reduced transcription of BMI_II339 which can be independently transcribed (Supplementary Figure S1).

### *Brucella* spp. Show Different Genotypic Profiles of the *gadBC–glsA* Locus

**FIGURE 5:**
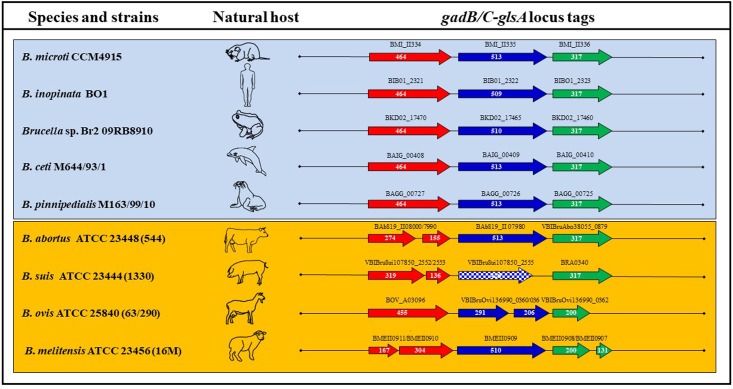
Schematic representation of the ORFs composing the *gadB/C*–*glsA* locus of representative *Brucella* species and strains. Each arrow length is proportional to the gene length, whereas the distance between two consecutive genes is not as precise, though reflecting the approximate distance between them. The corresponding locus tags are shown above each arrow. Squared arrows mark genes shorter than the canonical length of *B. microti* CCM4915. The host where each strain was first isolated is represented by a schematic picture.

The functionality of Gad (AR2) and AR2_Q systems in *B. microti* and the high degree of similarity among the genomes of *Brucella* spp. suggested the possible conservation of each or both of these systems among *Brucella* strains. In order to validate this hypothesis, a comparative analysis focused on the putative protein sequences of GadB, GadC, and GlsA (available from NCBI, Broad Institute, and PATRIC databases) was performed. Based on this analysis, these three proteins were predicted to be functional in new and atypical species/strains including *B. inopinata* BO1, *Brucella* sp. 83/13 isolated from Australian rodents, *Brucella* sp. BO2, *Brucella* sp. 09RB8471 and 09RB8910 isolated from frogs and marine mammal strains, i.e., *B. ceti* M644/93/1 and *B. pinnipedialis* M163/99/10 (**Figure 5** and Supplementary Table S1). Notably, the GlsA of the classical species *B. abortus* differs only by two amino acids from the *B. microti* counterpart and therefore could be potentially functional. With both GadC and GlsA potentially functional (Supplementary Table S1), *B. abortus* may have a functional AR2_Q system, though lacking a functional Gad system (Damiano et al., 2015). The presence of different types of mutations (frame-shifts, stop codons, etc.) in the coding sequences of *gadB*, *gadC*, and/or *glsA* suggested that none of the other classical species (*B. canis*, *B. melitensis*, *B. neotomae*, *B. ovis*, and all *B. suis* biovars) can be predicted to have functional AR2 systems.

### Only New and Atypical *Brucella* Spp. Have Functional Gad and AR2_Q Systems

**FIGURE 6:**
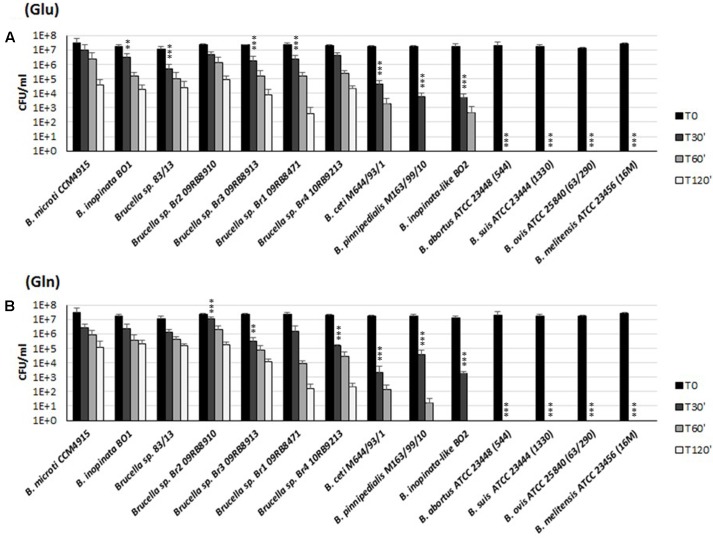
Survival of representative strains and species of *Brucella* during extreme acid exposure. Bacteria from a stationary phase cultures in TS at pH 7 were harvested and incubated in modified GMM at pH 2.5, supplemented with 3 mM of Glu **(A)** or Gln **(B)**. Residual viability following the acid shock was expressed as CFU/ml on a log10-scale at each time-point (T30, T60, and T120 min) and compared to unchallenged bacteria present at time T0 (GMM, pH 7.0). No viable bacteria were detected at the same time-points in the absence of Glu or Gln (data not shown). The data represent the mean (SD) of three independent experiments. Five strains of *B. abortus*, three strains of *B. suis*, two strains of *B. melitensis*, and one strain of *B. ovis*, *B. canis*, and *B. neotomae*, respectively (Supplementary Table S1), were also tested and found to be acid sensitive, as *B. abortus* ATCC 23488 (544), *B. suis* ATCC 23444 (1330), *B. ovis* ATCC 25840 (63/290), and *B. melitensis* ATCC 23456 (16 M) reported in the figure. A two-way ANOVA with a Bonferroni post-test statistical analysis was performed between *B. microti* (taken as reference for an acid-resistant *Brucella* species) and 13 representative *Brucella* species/strains (significance ^∗^*P* < 0.05, ^∗∗^*P* < 0.01, ^∗∗∗^*P* < 0.001).

To study the functionality of the AR2_Q system and its role in survival under extreme acid conditions, 26 representative strains of the genus *Brucella* (Supplementary Table S1) were tested for either Glu- or Gln-dependent AR. In agreement with the *in silico* analysis, the newly described strains of *Brucella* (including *B. microti* CCM4915, *B. inopinata* BO1, *Brucella* spp. 83-13, four *Brucella* strains isolated from African bullfrog and *Brucella* spp. BO2) and *Brucella* species isolated from marine mammals (*B. ceti* M644/93/1 and *B. pinnipedialis* M163/99/10) were able to survive in the presence of Gln for at least 30 min with a 10–10,000-fold reduction of viable bacteria, depending on the strain under analysis (**Figure 6B**). The AR of these strains in the presence of Glu was already published (Damiano et al., 2015), but re-performed in this work for comparative purposes and also because 3 mM L-Glu was used instead of 1.5 mM. As an outcome of this comparative work, we also observed a higher CFU (30–50%) recovery when Gln is used instead of Glu during the acid challenge of the wild-type strains (**Figures 1**, **3**, **4**, **6**), thus suggesting that GadB can contribute to the AR2_Q phenotype in *Brucella*, as previously suggested for *E. coli* and *L. reuteri* (Lu et al., 2013; Teixeira et al., 2014).

On the other hand and in agreement with their putatively non-functional Gad- and AR2_Q systems, *B. canis*, *B. melitensis*, *B. neotomae*, *B. ovis*, and *B. suis* biovars 1, 2, 3, and 4 were not recovered already after 30 min at pH 2.5 in the presence of either Glu or Gln (**Figure 6** and data not shown).

**FIGURE 7:**
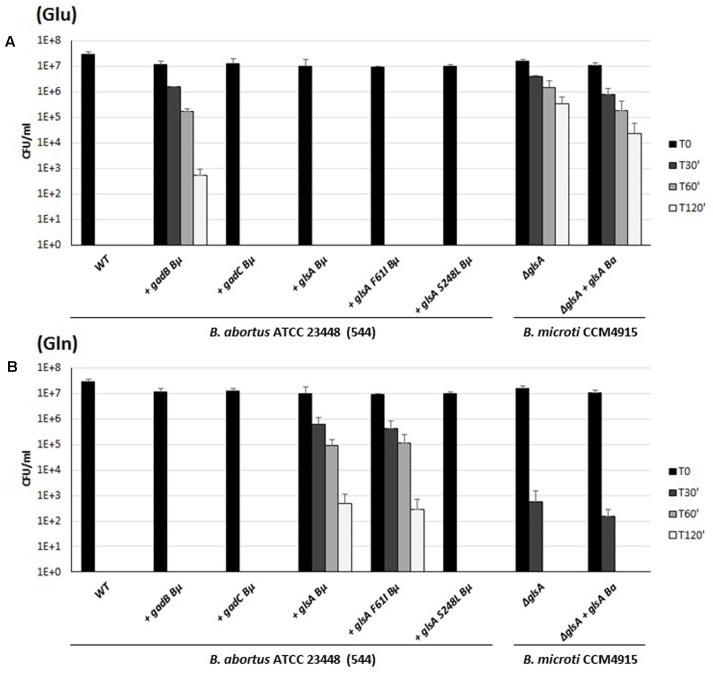
Survival of wild-type, *B. abortus* ATCC 23448 and *B. microti* CCM4915 mutant and complemented strains during extreme acid exposure. Bacteria from stationary-phase cultures in TS at pH 7 and 5% CO_2_ were harvested and incubated in a modified GMM at pH 2.5 supplemented with 3 mM of Glu **(A)** or Gln **(B)**. Heterologously complemented strains are marked with a “+” followed by the designation of the complementing gene, and the acronym of the donor species, i.e., *B*μ for *B. microti* and *Ba* for *B. abortus*. Two strains of *B. abortus* express *in trans* a modified version of the GlsA protein of *B. microti* with amino acid substitutions Phe61Ile or Ser248Leu. Residual viability following the acid shock was expressed as CFU/ml on a log10-scale at each time-point (T30, T60, and T120 min) and compared to unchallenged bacteria present at time T0 (GMM, pH 7.0). No viable bacteria were detected at the same time-points in the absence of Glu or Gln (data not shown). The data represent the mean (SD) of three independent experiments.

Sequence analysis of *gadB* of *B. abortus* revealed a stop codon in the first third of the gene, resulting in a putative truncated protein of 155 amino acids (Supplementary Table S1). This most likely explained the Gad-negative phenotype, as well as the acid-sensitive phenotype in the presence of Glu (Damiano et al., 2015). Despite their potentially functional GadC and GlsA proteins, six strains belonging to different biovars of *B. abortus* were also found to be acid-sensitive in the presence of Gln (**Figure 6B** and data not shown). To investigate the reason of the acid sensitivity of *B. abortus*, individual *gadB*, *gadC*, and *glsA* genes of *B. microti* were expressed *in trans* in the *B. abortus* ATCC 23448 strain (**Figure 7**). The *B. abortus* derivative strains carrying *in trans gadB* and *glsA*, but not *gadC* of *B. microti*, were able to survive in the presence of Glu and Gln, respectively. On the contrary, the Δ*glsA*-mutant strain of *B. microti* did not recover the Gln-dependent AR phenotype when was complemented with *glsA* of *B. abortus* (**Figure 7**, two rightmost sets of bars). This suggested that the GadC antiporter is operative, whereas both GadB (as already reported) and GlsA are not functional in this species (**Figure 7**).

**FIGURE 8:**
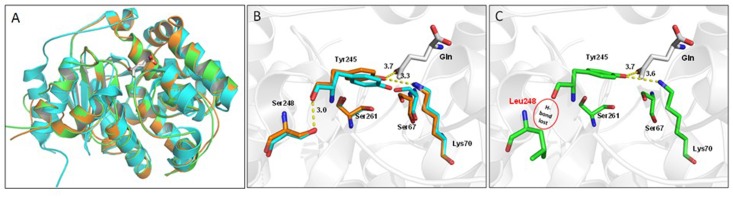
Structural comparison of glutaminase from *Escherichia coli* (YbaS, 1U60, cyan cartoon), *B. microti* CCM4915 (orange), and *B. abortus* ATCC 23448 (544) (green). **(A)** Superimposition of the three glutaminases, the substrate Gln is depicted as white thick sticks and enzyme secondary elements are shown in cartoon representation. **(B)** Close-up view of the active site of YbaS from *E. coli* and GlsA from *B. microti* showing key residues involved in the substrate-binding site. Residue numbering refers to *B. microti*, with Ser67, Lys70, Tyr245, Ser248, and Ser261 corresponding to the *E. coli* counterparts Ser66, Lys69, Tyr244, Ser247, and Ser260, respectively (Brown et al., 2008). The network of hydrogen bonds including that between Ser248 and Tyr245 a residue connected to Gln is shown (all distances are labeled in Å). **(C)** Same as in B for *B. abortus* with Ser248Leu mutation indicating the absence of a hydrogen bond (highlighted by a red circle) and a destabilization of the rest of the network.

Sequence comparisons of all putative GlsA proteins of *B. abortus* (i.e., 234 sequences available at the PATRIC genome website at the time of writing of this paper) with *B. microti* GlsA revealed the systematic replacement of two amino acids, namely Phe61→Ile and Ser248→Leu. Therefore, *Brucella* glutaminases model structures were obtained by comparative homology modeling (**Figure 8A**). The single amino acid substitution Ser248Leu in *B. abortus* attracted our attention as this residue is located not far from the Gln-binding site (**Figure 8B**). Indeed, glutaminases from *E. coli* and *B. microti* own a serine residue at this position which is directly connected through hydrogen bonding to one of the residues that in *E. coli* and *B. subtilis* glutaminases was shown to be actively participating in catalysis, i.e., Tyr244 in *E. coli* [corresponding to Tyr245 in *B. microti* (Brown et al., 2008)]. Molecular modeling suggested that this hydrogen bond is lacking in *B. abortus* where Ser248 is replaced with a Leu residue (**Figure 8C**). To validate the correctness of the modeling predictions, two copies of *glsA* of *B. microti*, each containing only one of the two point mutations found in the *B. abortus* gene, were generated and introduced into *B. abortus* ATCC 23448 for *in trans* complementation assays. As predicted by the modeling, the *B. abortus* strain expressing from a muticopy plasmid the GlsA_Ser248Leu variant of *B. microti* remains hypersensitive to acid stress in GMM pH 2.5 in the presence of Gln. In contrast, *B. abortus* expressing either the wild type or the GlsA_Phe61Ile variant of *B. microti* had the AR phenotype restored (**Figure 7**). These results clearly indicated that in *B. abortus* the Ser248Leu substitution in the GlsA enzyme is responsible for the loss of enzyme activity, likely due to an incorrect positioning of Tyr245 and other catalytically important residues in the active site of the protein, as depicted in **Figure 8C**.

## Discussion

In unicellular organisms, the presence of mechanisms protecting against drastic, sudden, and/or prolonged exposure to an acidic environment is clearly beneficial for the proper functioning of the metabolic enzymes, the preservation of the transmembrane potential, and therefore cellular viability and, eventually, fitness. In foodborne pathogenic bacteria, this is also regarded as a pre-requisite for host infection (De Biase and Pennacchietti, 2012; Lund et al., 2014).

*Brucella* spp. may experience acid stress in different environments like certain soils, fermented food, some districts of the gastro-intestinal tract of the hosts, and the intracellular vacuole (Porte et al., 1999; Sangari et al., 2007; Falenski et al., 2011). In previous reports we have shown that *B. microti*, new species and atypical strains of *Brucella*, and isolates from marine mammals possess a Gad system functionally homologous to that of *E. coli* (Occhialini et al., 2012; Grassini et al., 2015). This system was also shown to protect *B. microti* not only *in vitro* at pH 2.5 in the sole presence of Glu, but also *in vivo* following an oral route of infection in a murine model (Occhialini et al., 2012). More recently, in *E. coli* and *L. reuteri*, physiological and genetic approaches allowed to demonstrate that the antiporter GadC, a structural component of the Gad system, can also work together with a glutaminase (YbaS in *E. coli* or Gls3 in *L. reuteri*), as part of a Gln-dependent AR system (named AR2_Q) (Su et al., 2011; Lu et al., 2013; Lund et al., 2014; Teixeira et al., 2014).

We noticed that immediately downstream the *gadB* and *gadC* genes of the Gad system, a *glsA* gene, coding for a putative glutaminase, and an *hdeA* gene, coding for a periplasmic chaperone (Valderas et al., 2005), occur in all *Brucella* genomes so far sequenced, thus raising the hypothesis that they might constitute an operon and participate in AR. This hypothesis was supported by our finding that among the 20 proteinogenic amino acids, *B. microti* displays a pH 2.5-AR phenotype only in the presence of Glu or Gln (**Figure 1**).

Herein, we show in this work by genetic and molecular approaches that *glsA* and *gadC* code for the structural determinants of the AR2_Q system in *B. microti*.

Among the genes (i.e., *hdeA*, BMI_II338 and BMI_II339) that were found to be co-transcribed with *gadB*, *gadC*, and *glsA* (**Figure 2** and Supplementary Figure S1), only BMI_II339 contributes to Glu- and Gln-dependent AR (**Figure 3**). Moreover, the independent transcription of *hdeA* and BMI_II339, assessed by RNAseq (Supplementary Figure S1), suggested that these two genes can be regulated differently.

Depending on the source database (Patric or NCBI) the sequence of BMI_II339 is predicted to be either 83 or 54 amino acids long. Despite the difference in length of the N-terminal portion of BMI_339 and its homologs in other *Brucella* species (Supplementary Figure S2A), the secondary structure predictions suggest that this protein possesses two transmembrane domains separated by four to five amino acids. Notably, the first transmembrane segment of BMI_II339 shares 42.8/61.9% identity/similarity with the single transmembrane segment in the “connector” SafA (Supplementary Figure S2) involved in the modulation of the acid stress response in *E. coli* (Mitrophanov and Groisman, 2008). This may suggest that BMI_II339 could also act as connector affecting the activity of an acid sensing apparatus that in *Brucella* has not yet been discovered, in contrast to *E. coli* (Eguchi et al., 2012). On the other hand, BMI_II339 may be a member of bacterial transmembrane proteins that lack N-terminal signal sequences (Craney et al., 2011). At present we can only speculate on the role of this protein, however, the finding that the *in trans* complementation of the *B. microti* ΔBMI_II339 mutant with plasmid pBBR1-MCS carrying a copy of the same gene restores the Glu- and Gln-dependent AR phenotype suggests a genuine involvement of this small protein in AR also in the “classical” Brucellae where the protein sequence is conserved and intact (Supplementary Figure S2A). Mutagenesis and gene expression studies will be required to assess the role of BMI_II339.

The question how and by which stimulus the *gadBC–glsA* operon may be activated remains yet unanswered. However, because the mutations in the Gad and AR2_Q structural genes seem to affect their translation rather than the transcription, possible hints could come from previously performed global transcriptome analyses of the most-studied classical species *B. abortus*, *B. melitensis*, and *B. suis.* For example, in these species the expression of *gadB*, *gadC*, *glsA*, and *hdeA* was found to be controlled by major regulators such as MucR, Rsh, and Hfq (Caswell et al., 2013; Cui et al., 2013; Hanna et al., 2013).

As part of this work, we aimed at deepening our understanding of the conservation of the AR2_Q system among the *Brucella* genus. Using a large panel of *Brucella* strains, we found that in addition to the Gad system (Damiano et al., 2015), the AR2_Q system is operative in new and atypical species, and isolates from marine mammals, whereas both systems are not functional in all the classical species. Notably, in *B. abortus*, a highly pathogenic classical species, the AR2_Q system was predicted to be functional. However, the constant occurrence of the same two amino acid substitutions (i.e., Ser248→Leu and Phe61→Ile) in the GlsA of all sequenced strains of this species, respect to *B. microti*, suggested that at least one of the two is at the origin of the observed lack of AR2_Q. The Ser248Leu substitution attracted our attention and by site-directed mutagenesis driven by modeling studies, we confirmed that residue Ser248 plays an important role in the deamidation reaction, probably by stabilizing the enzyme catalytic core and allowing the correct positioning of Tyr245 involved in the Gln binding (**Figure 8**) (Brown et al., 2008). Therefore, with this work we contributed to highlight the catalytically important role of Ser248, a residue previously unidentified during a systematic mutagenesis and structural study of the glutaminases from *E. coli* and *B. subtilis* (Brown et al., 2008).

The major unresolved issue is to understand why only the new species and atypical strains of *Brucella* maintain these two AR2 systems functional, whereas classical terrestrial strains have lost them, despite their well-known oral route of infection in mammalian hosts, humans included. Our data suggest that in the most ancient strains of *Brucella*, both AR2 systems are functional and in the classical species these functions were lost during their pathoadaptive evolution (Merhej et al., 2013). One very fine example is provided herein by the finding that the Ser248Leu mutation found in *B. abortus* is sufficient to abolish the AR2_Q phenotype in this species, without the loss of a gene. On the other hand, the consequences of the loss of functionality of these systems can be considered as neutral or negative with respect to bacteria–host interaction. Indeed, no differences in the growth rates and in survival were observed between the wild-type and the *gadB/C-* and *glsA*-mutant strains (i.e., without Gad- or AR2_Q-systems) of *B. microti in vitro* in TS medium, under intermediate acid stress (GMM medium at pH 4.5) and during experimental host cell infection (Occhialini et al., 2012 and Supplementary Figure S3).

Our data suggest that the functional state of the Gad- and AR2_Q-systems may reflect the adaptive evolution of *Brucella* species to their respective environments and hosts. During their evolutionary adaptation to specific hosts, however, the classical *Brucella* species with the exception of *B. ovis* retained a functional urease-dependent AR system. This system was described as essential for the success of oral infection in *B. suis*, *B. abortus*, and *B. melitensis* (Bandara et al., 2007; Sangari et al., 2007; Paixão et al., 2009) as well as in other pathogenic orally acquired bacteria, such as *Helicobacter pylori* and *Yersinia enterocolitica* (Marshall et al., 1990; Young et al., 1996). As urea is very abundant in the stomach, the urease system is likely sufficient for the survival of these species. On the other hand, while new and atypical strains are described as urease-positive, no through studies have yet been carried out concerning the role of the urease system in protection from acid stress. It would therefore be interesting to explore the contribution of the urease system in these strains with respect to their adaptation to acidic conditions.

The question whether the group of newly discovered strains, but evolutionary more ancestral, as compared to the group of the classical terrestrial species, are armed with a whole range of systems protecting from acid stress (from mild to extreme) is still an open issue of great interest that deserves to be further explored as it might be linked to fitness in different niches, depending on the available substrates.

A practical outcome of this work could be the development of new biochemical diagnostic tests based on Gad and GlsA activities helping in assigning the membership of new *Brucella* isolates to either one or the other group.

## Author Contributions

AO, DB, and SK designed the study. LF, MD, and AO carried out the experiments and the analysis of data with *Brucella*; EP and DB with *E. coli*. SAD performed phenotyping of the bullfrog strains of *Brucella*. LC executed protein modeling. LC and DB performed bioinformatics analysis. AO, DB, LF, and SK were involved in drafting the manuscript and all authors read and approved the final manuscript.

## Conflict of Interest Statement

The authors declare that the research was conducted in the absence of any commercial or financial relationships that could be construed as a potential conflict of interest.

## Footnotes

1 eurofinsgenomics.com

2 www.expasy.org/proteomics
