# Supplementary material for: The Glutaminase-Dependent System Confers Extreme Acid Resistance to New Species and Atypical Strains of Brucella
Source: Front Microbiol. 2017 Nov 15;8:2236. doi: 10.3389/fmicb.2017.02236 (PMC5695133; doi:10.3389/fmicb.2017.02236)
Supplement: Supplementary file 1 [file Table_1.PDF]

Supplementary Material

The glutaminase-dependent system confers extreme acid resistance to new species and atypical strains of *Brucella*

Luca Freddi, Maria Alessandra Damiano, Laurent Chaloin, Eugenia Pennacchietti, Sascha Al Dahouk, Stephan Köhler, Daniela De Biase and Alessandra Occhialini[*]

*** Correspondence:** Alessandra Occhialini: alessandra.occhialini@irim.cnrs.fr

Supplementary Table 1: Sizes and genetic coordinates of GadB, GadC and GlsA proteins of *Brucella* spp.

**Species and Strains**	**Other reference**	**Serotype**	**Origin**	**GadB[1]**	**GadB PATRIC ID or RefSeq locus tag**	**GadC[1]**	**GadC PATRIC ID or RefSeq locus tag**	**GlsA[1]**	**GlsA PATRIC ID or RefSeq locus tag**
B. abortus									
ATCC 23448 (544)*	NCTC10093	1	Cattle	155/304	B977_02994/02993	510	B977_02992	317	B977_02991
ATCC 23450 (Tulya)*	NCTC10502	3	Cattle	145/286	VBIBruAbo20544_3082/3083	510	BACG_00413	317	BACG_00414
ATCC 23451 (292)*	NCTC10503	4	Cattle	155/286	VBIBruAbo93713_2600/2599	513	BABG_00908	317	BABG_00907 DK59_895
ATCC 23452 (B3196)*	NCTC10504	5	Cattle	145/286	VBIBruAbo58081_2541/2540	510	BAYG_02558	317	BAYG_02557
ATCC 23453 (870)*	NCTC10505	6	Cattle	155/286	VBIBruAbo118911_3398/3397	513	BAAG_00701	317	BAAG_00700
ATCC 23455 (C68)*	NCTC10507	9	Cattle	155/286	VBIBruAbo122389_1123/1122	513	BARG_00691	317	BARG_00690
B. canis									
ATCC 23365 (RM 6/66)*	NCTC10854	1	Dog	319/136	BCAN_B0340	48/426	BCAN_B0341	317	BCAN_B0342
B. ceti									
L2/15 (BCCN98-54)*	M644/93/1		Common dolphin	464	BAIG_00408	513	BAIG_00409	317	BAIG_00410
B1/94 (BCCN94-74)	NCTC 12891	1	Porpoise	464	BAQG_02233	513	BAQG_02234	317	BAQG_02235
L2/39 (BCCN98-78)	M490/95/1		Common seal	464	BAPG_02267	513	BAPG_02266	317	BAPG_02265
B. inopinata									
BO1*	CDC San.Ak.BW		Human	464	BIB01_2321	509	BIB01_2322	317	BIBO1_2323
***B. inopinata*-like**									
BO2*	CDC		Human	464	BIB02_0627	471	BIB02_0626	317	BIBO2_0625
B. melitensis									
ATCC 23456 (16M)*	NCTC10094		Goat	159/286	BMEII0911/ 0910	510	BMEII0909	200/131	BMEII0908/ 0907
ATCC 23457 (63/9)*	NCTC10508		Goat	150/286	BMEA_B0338	521	BMEA_B0339	200/131	BMEA_B0340
ATCC 23458 (Ether)*	NCTC10509	3	Goat	159/286	VBIBruMel115656_0303/0302	490	DK62_3067	200/131	DK62_3066/ 3065
B. microti									
CCM4915 BCCN07-01*			Common vole	464	BMI_II334	510	BMI_II335	317	BMI_II336

Species and Strains	Other reference	Serotype	Origin	GadB[1]	GadB PATRIC ID or RefSeq locus tag	GadC[1]	GadC PATRIC ID or RefSeq locus tag	GlsA[1]	GlsA PATRIC ID or RefSeq locus tag
B. neotomae									
5K33*	NCTC10084		Desert wood rat	263/201	VBIBruNeo114381_0326/0325	513	BANG_02613	317	BANG_02612
B. ovis									
ATCC 25840 (63/290)*	NCTC10512	1	sheep	455	BOV_A0309	291/206	BOV_A0310	200/131	BOV_A0311
B. pinnipedialis									
BCCN06-44*	M163/99/10		Hooded seal	464	BAGG_00727	513	BAGG_00726	317	BAGG_00725
B. suis									
ATCC 23444 (1330)*	NCTC10316	1	Swine	319/136	BRA0338	426/84	BRA0339	317	BRA0340
ATCC 23445 (Thomsen, BCCN-R13)*	NCTC10510	2	Swine	454	BSUIS_B0344	336	BSUIS_B0345	317	BSUIS_B0346
ATCC 23446 (686)*	NCTC10511	3	Swine	309/136	VBIBruSui102552_0231/0232	336	VBIBruSui102552_0234	46/272	VBIBruSui102552_0235/236
ATCC 23447 (40)*	NCTC11364	4	Reindeer	310/136	VBIBruSui38587_3116/3115	486	BAVG_2470	317	BAVG_2469
513	NCTC11996	5	Wild rodent	295/165	VBIBruSui73489_0123/0124	513	BAEG_02298	187/128	VBIBruSui73489_0127/0128
Brucella sp.									
83/13*			Australian rodent	464	BAKG_02483	513	BAKG_02482	317	BAKG_02481
09RB8471*	Br1		African bullfrog	464	BKD03_02495	509	BKD03_02500	317	BKD03_02505
09RB8910*	Br2		African bullfrog	464	BKD02_17470	510	BKD02_17465	317	BKD02_17460
09RB8913*	Br3		African bullfrog	ND	ND	ND	ND	ND	ND
10RB9213*	Br4		African bullfrog	ND	ND	ND	ND	ND	ND
E. coli									
K12 MG1655				466	b3516 and b1493	511	b1492	310 and 308	b0485 and b1524

* Bacterial strains studied in vitro in this work; ND: not determined (sequence unavailable). [1]: Number of amino acids. "/" indicates presence of a Stop codon at the origin of the two fragments of the indicated sizes.
