# Supplementary material for: The Glutaminase-Dependent System Confers Extreme Acid Resistance to New Species and Atypical Strains of Brucella
Source: Front Microbiol. 2017 Nov 15;8:2236. doi: 10.3389/fmicb.2017.02236 (PMC5695133; doi:10.3389/fmicb.2017.02236)
Supplement: Supplementary file 2 [file Table_2.PDF]

Supplementary Material

The glutaminase-dependent system confers extreme acid resistance to new species and atypical strains of *Brucella*

Luca Freddi, Maria Alessandra Damiano, Laurent Chaloin, Eugenia Pennacchietti, Sascha Al Dahouk, Stephan Köhler, Daniela De Biase and Alessandra Occhialini*

*** Correspondence:** Alessandra Occhialini: alessandra.occhialini@irim.cnrs.fr

Supplementary Table S2: Primers used in this study for genetic manipulations and RT-PCR analysis

Use	Name	Primer sequence 5'-3'	Target gene	Fragment dimension	Union-PCR fragment dimension	Restriction enzyme
Mutagenesis	ΔgadB_For	TAGCGTCAGTTACGTATCGAAG	*ΔgadB*	1666 bp	-	*BspI*
	ΔgadB_Rev	AAGCGTAAGCACACTAAGCTTC				
	ΔgadC_For	GGTTATTCGCTCTATGATCTGTC	*ΔgadC*	1907 bp	-	*ClaI*
	ΔgadC_Rev	TTTGATCGCATCTGATGAGCTTG				
	ΔglsA_For	CCTTTCTTCCAGAAGGTGAATGC	*ΔglsA*	2016 bp	-	*StuI*
	ΔglsA_Rev	AGGCAGCGGCGAAG				
	ΔHdeA_For	AATGCAGGCGCAATTGCAACTGC	*ΔhdeA*	1828 bp	-	*BbsI*
	ΔHdeA_Rev	CAGAAGGTAACGGCAAGTGCCAG				
	gadBC-op_PstI-For	GCCCTGCAGCCGAGCTTATTGCGCTAATATC	*ΔgadB/C-glsA*	4713 bp	-	*ClaI/HindIII*
	hdeA_RT_Rev	TTAAAGCCTACGACCCAACC				
	Master_glsA_XhoI_For	GCCCTCGAGGAAAAGCCCGTCCTGCAAAAGC	mutation F61I *glsA Bμ*	237 bp	1031 bp	*XhoI/XbaI*
	M61-glsA-Union-For	CGATGCCGATATCGCCTTCGCTATCGAGTC				
	M61-glsA-Union-Rev	GACTCGATAGCGAAGGCGATATCGGCATCG		794 bp		
	Master_glsA_XbaI_Rev	GCCTCTAGAGAGACCTTCCTCAGGATGAC				
	Master_glsA_XhoI_For	GCCCTCGAGGAAAAGCCCGTCCTGCAAAAGC	mutation S248L *glsA Bμ*	797 bp	1032 bp	*XhoI/XbaI*
	M248-glsA-Union-For	CTTTATACGGCTTTAGGCGATTGGGCTTATAC				
	M248-glsA-Union-Rev	GTATAAGCCCAATCGCCTAAAGCCGTATAAAG		235 bp		
	Master_glsA_XbaI_Rev	GCCTCTAGAGAGACCTTCCTCAGGATGAC				
	$Kana^R$_For	GAGCTTTGTTGTAGGTGGAC	$Kana^R$ cassette	1207 bp	-	-
	$Kana^R$_Rev	TGGAATTGTGAGCGGATAAC				
Complementation	gadB_XhoI_Comp_For	CCGCTCGAGTAGCGTCAGTTACGTATCGAAG	Comp_*gadB*	1666 bp	-	*XhoI/XbaI*
	gadB_XbaI_Comp_Ref	GGATCTAGAAAGCGTAAGCACACTAAGCTTC				
	gadC_XhoI_Comp_For	CCGCTCGAGCACTCTTCATTCGGGATTTCAAG	Comp_*gadC*	1768 bp	-	*XhoI/XbaI*
	gadC_XbaI_Comp_Ref	GGATCTAGATTTGATCGCATCTGATGAGCTTG				
	glut_Comp_XhoI_For	GCCCTCGAGGAAAAGCCCGTCCTGCAAAAGC	Comp_*glsA*	1001 bp	-	*XhoI/XbaI*
	glut_Comp_XbaI_Rev	GCCTCTAGAGAGACCTTCCTCAGGATGAC				
	hdeA_Comp_XhoI_For	GCCCTCGAGTGTCATCCTGAGGAAGGTCTCGG	Comp_*hdeA*	519 bp	-	*XhoI/XbaI*
	hdeA_Comp_XbaI_Rev	CGGTCTAGAAGGCAGCGGCGAAGGATCAG				
	gadBC-op_PstI-For	GCCCTGCAGCCGAGCTTATTGCGCTAATATC	Comp_*gadB/C*	3503 dp	-	*PstI/XbaI*
	gadBC-op_XbaI-Rev	GCCTCTAGAATCGCATCTGATGAGCTTGAC				
	gadC_XhoI_Comp_For	CCGCTCGAGCACTCTTCATTCGGGATTTCAAG	Comp_*gadC-glsA*	2885 bp	-	*XhoI/XbaI*
	glsA_XbaI_Comp_Ref	GGATCTAGACGGCTGCAAGAGCAGTATTC				
Coexpression in operon (RT-PCR)	BMI_II333 for	ATTTTAGTTCCCGTGAAAGC	*BMI_II333-gadB*	438 bp	-	-
	BMI_II334 rev	GTCGATCCAGTTCTTGTTGA				
	BMI_II334 for	CACTCTTCATTCGGGATTTCAAG	*gadB-gadC*	387 bp	-	-
	BMI_II335 rev	CGACAATCGATACGGGAATAAG				
	BMI_II335 for	TGATTTTCGTCGCTATCCCATTG	*gadC-glsA*	493 bp	-	-
	BMI_II336 rev	TTCGAGCGCGATTACAGAATTG				
	BMI_II336 for	AATAGTGTGAAAGCAATGGCTGC	*glsA-hdeA*	368 bp	-	-
	BMI_II337 rev	AGTTCACTCACCTTCTTCTTTG				
	BMI_II337 for	TAAGAATACTGCTCTTGCAGCC	*hdeA-BMI_II338*	534 bp	-	-
	BMI_II338 rev	TAACGGATAACCAAGCTCAGG				
	BMI_II338 for	CCTGAGCTTGGTTATCCGTTA	*BMI_II338-BMI_II339*	368 bp	-	-
	BMI_II339 rev	TCGCGCCACTTGCTTCCCACG				
	BMI_II338 for	CCTGAGCTTGGTTATCCGTTA	*BMI_II338-EAL dcp*	760 bp	-	-
	BMI_II340 rev	CTCTATTCCCCACCGCTTC				
