# Supplementary material for: The Glutaminase-Dependent System Confers Extreme Acid Resistance to New Species and Atypical Strains of Brucella
Source: Front Microbiol. 2017 Nov 15;8:2236. doi: 10.3389/fmicb.2017.02236 (PMC5695133; doi:10.3389/fmicb.2017.02236)
Supplement: Supplementary file 3 [file Image_1.PDF]

Supplementary Material

The glutaminase-dependent system confers extreme acid resistance to new and atypical species/strains of *Brucella*

Luca Freddi, Maria Alessandra Damiano, Laurent Chaloin, Eugenia Pennacchietti, Sascha Al Dahouk, Stephan Köhler, Daniela De Biase and Alessandra Occhialini*

*** Correspondence:** Alessandra Occhialini: alessandra.occhialini@irim.cnrs.fr

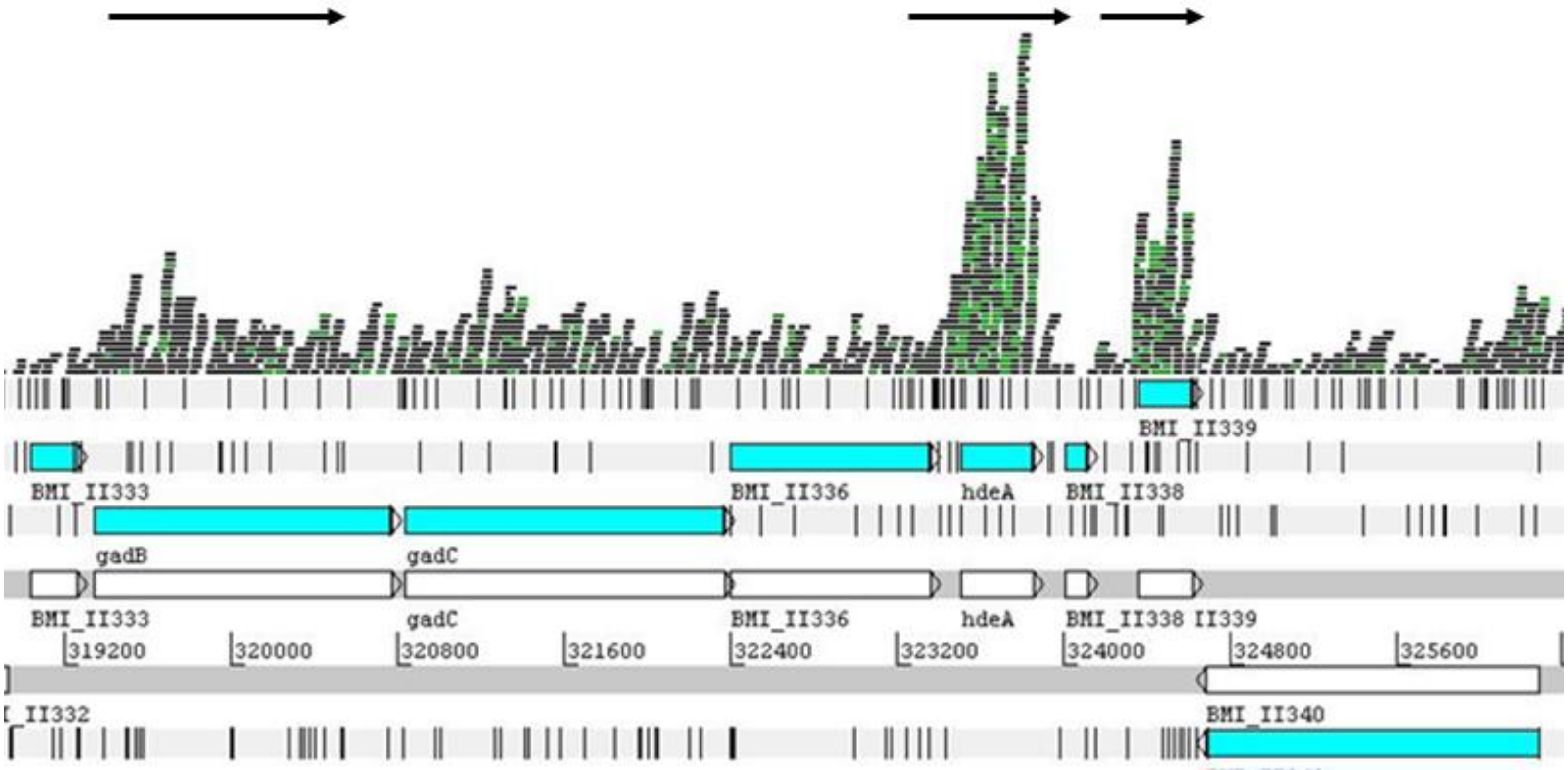

Figure S1. The read alignment view using Artemis software (Sanger Institute, UK) showing RNA-Seq data for the region including the *gadB/C-glsA* locus of *B. microti* CCM4915 on chromosome II. The arrows above the reads show the three major transcripts in the locus.
