# Supplementary material for: The Glutaminase-Dependent System Confers Extreme Acid Resistance to New Species and Atypical Strains of Brucella
Source: Front Microbiol. 2017 Nov 15;8:2236. doi: 10.3389/fmicb.2017.02236 (PMC5695133; doi:10.3389/fmicb.2017.02236)
Supplement: Supplementary file 4 [file Image_2.PDF]

Supplementary Material

The glutaminase-dependent system confers extreme acid resistance to new species and atypical strains of *Brucella*

Luca Freddi, Maria Alessandra Damiano, Laurent Chaloin, Eugenia Pennacchietti, Sascha Al Dahouk, Stephan Köhler, Daniela De Biase and Alessandra Occhialini*

*** Correspondence:** Alessandra Occhialini: alessandra.occhialini@irim.cnrs.fr

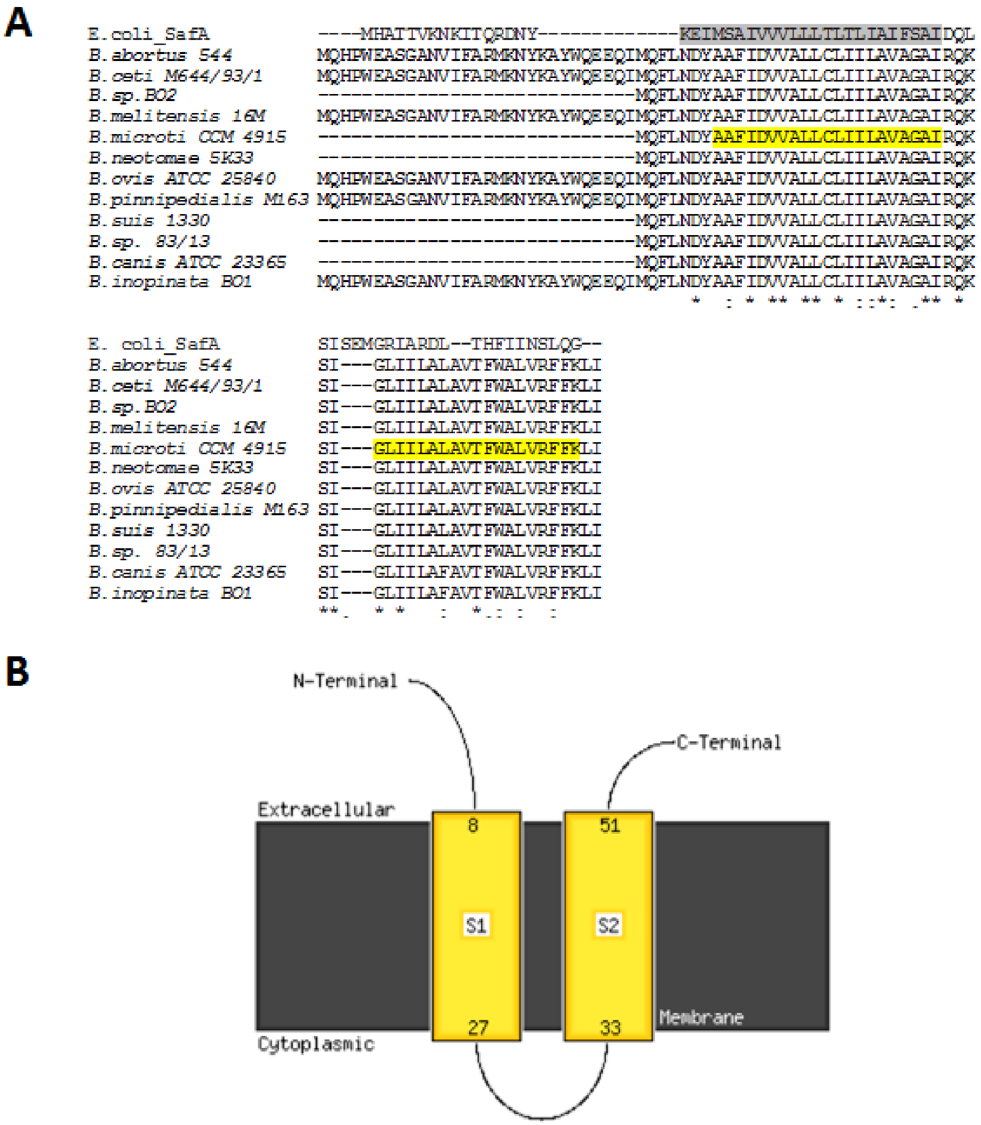

Figure S2. **Prediction of secondary structure and membrane localization of BMI_II339, involved in AR2 systems in *B. microti* CCM4915. A)** Clustal Omega multiple sequence alignments of BMI_II339 putative proteins from representative strains of *Brucella* (selected from PATRIC database). The *E. coli* SafA protein is also aligned for comparing the identity/similarity with BMI_II339. The sequence in gray background refers to the transmembrane domain in *E. coli* SafA, whereas the sequences in yellow background refer to the predicted two transmembrane domains in *B. microti* BMI_II339. **B)** Secondary structure, membrane localization and orientation of BMI_II339 as predicted by MEMSAT (bioinf.cs.ucl.ac.uk/psipred/).
