# Supplementary material for: The Glutaminase-Dependent System Confers Extreme Acid Resistance to New Species and Atypical Strains of Brucella
Source: Front Microbiol. 2017 Nov 15;8:2236. doi: 10.3389/fmicb.2017.02236 (PMC5695133; doi:10.3389/fmicb.2017.02236)
Supplement: Supplementary file 5 [file Image_3.PDF]

Supplementary Material

The glutaminase-dependent system confers extreme acid resistance to new and atypical species/strains of *Brucella*

Luca Freddi, Maria Alessandra Damiano, Laurent Chaloin, Eugenia Pennacchietti, Sascha Al Dahouk, Stephan Köhler, Daniela De Biase and Alessandra Occhialini*

*** Correspondence:** Alessandra Occhialini: alessandra.occhialini@irim.cnrs.fr

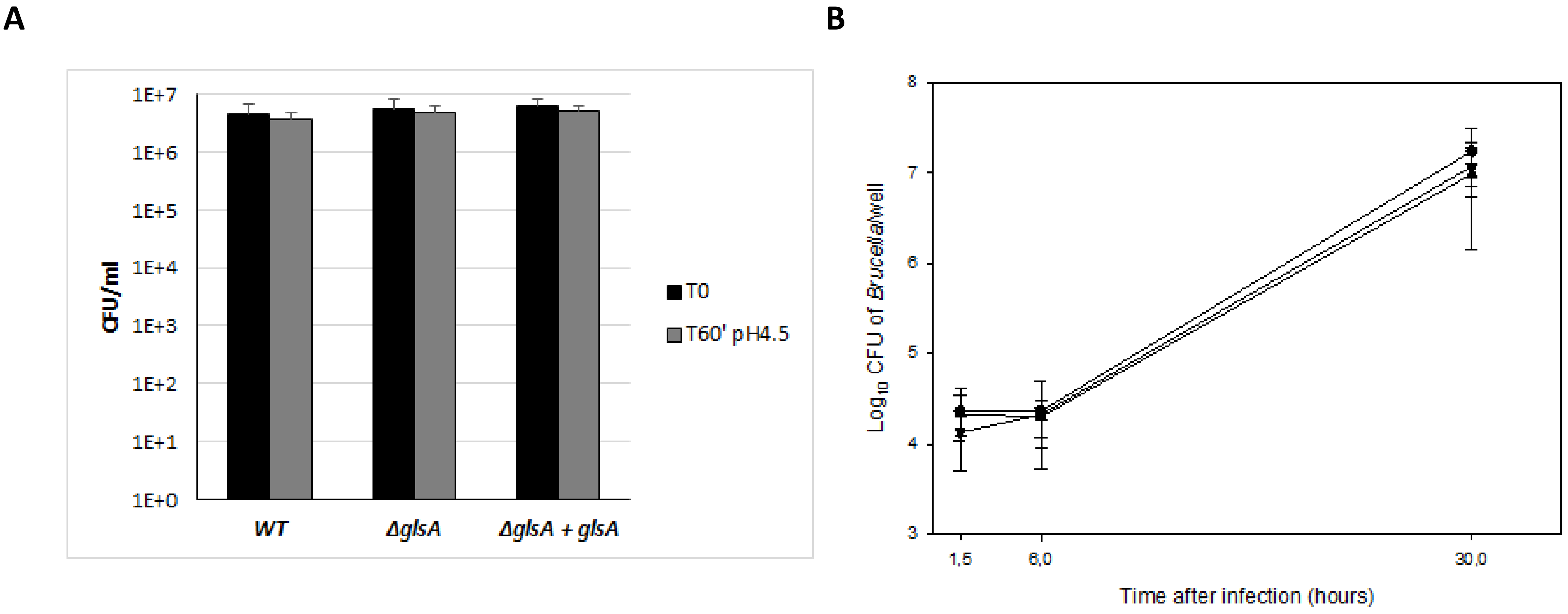

Figure S3. Survival of wild-type (WT), Δ*glsA* and Δ*glsA*+*glsA* mutant strains of *Brucella microti* CCM4915 *in vitro* (A) and *in cellulo* (B). A) Bacteria from stationary-phase cultures in TS at pH 7 were harvested and incubated for 60 min in modified GMM at pH 7 and 4.5 supplemented with 3 mM of Gln. **B)** Intracellular multiplication in J774 murine macrophagic cells were carried out using the protocol published previously by our group (Jiménez de Bagüés et al., 2010). Data points represent the mean (SD) of three independent experiments. No significant differences were found at any time point between the strains recovered from acid stress at pH 4.5 and from infected cells, respectively. Symbol of strains: wild-type (●), Δ*glsA* (▼), Δ*glsA* complemented with *glsA* of *B. microti* (▲).
